# Cardiovascular risk factor burden in Africa and the Middle East across country income categories: a post hoc analysis of the cross-sectional Africa Middle East Cardiovascular Epidemiological (ACE) study

**DOI:** 10.1186/s13690-018-0257-5

**Published:** 2018-02-12

**Authors:** Frederick J. Raal, Alawi A. Alsheikh-Ali, Mohamed I. Omar, Wafa Rashed, Omar Hamoui, Abdoul Kane, Mohamed Alami, Paula Abreu, Walid M. Mashhoud

**Affiliations:** 1 Carbohydrate & Lipid Metabolism Research Unit, Division of Endocrinology & Metabolism, Department of Medicine, Faculty of Health Sciences, Johannesburg Hospital, University of the Witwatersrand, Parktown, Johannesburg, 2193 South Africa; 2 College of Medicine, Mohammed Bin Rashid University of Medicine and Health Sciences, Dubai, United Arab Emirates; 3 Pfizer Gulf FZ LLC, Dubai Media City, Dubai, United Arab Emirates; 4 Cardiology Division, Mubarak Al-Kabeer Hospital, Kuwait City, Kuwait; 5 Clemenceau Medical Center, Cardiovascular Diseases, Beirut, Lebanon; 6 Department of Cardiology, Dakar University, Hospital General de Grand Yoff, Dakar, Senegal; 7 Private Practice, Casablanca, Morocco; 8 Pfizer Inc, New York, NY USA; 9 Pfizer Saudi Limited, Jeddah, Saudi Arabia

**Keywords:** Cardiovascular risk, Developing countries, Epidemiology, Africa, Middle East

## Abstract

**Background:**

A significant number of cardiovascular disease (CVD)-related deaths occur in developing countries. An increasing prevalence of CVD is associated with a change in the macro-economy of these countries. In this post hoc analysis, CVD risk factor (CVDRF) prevalence is evaluated across countries based on national income in the Africa and Middle East Region (AfME).

**Methods:**

Data from the Africa Middle East Cardiovascular Epidemiological (ACE) study were used; a cross-sectional study in 14 AfME countries (94 clinics) from July 2011–April 2012, which evaluated CVDRF prevalence in stable adult outpatients. World Bank definitions were used to classify countries as low-income (LI), lower-middle-income (LMI), upper-middle-income (UMI) or high-income (HI) countries. Four thousand three hundred seventy-eight subjects were recruited where 260 (6%), 1324 (30%), 1509 (35%) and 1285 (29%) were from LI, LMI, UMI, and HI countries, respectively.

**Results:**

Of all the CVDRFs evaluated, almost two-thirds of the study population across the national income groups had abdominal obesity and dyslipidemia. Countries in the HI category were associated with a higher prevalence of diabetes (32%), obesity (44%) and smoking (16%). UMI and HI countries were associated with higher clustering of CVDRFs where at least one-third of subjects having four or more CVDRFs. Lower income countries had lower blood pressure control rates and lower percentages of outpatients achieving LDL-cholesterol targets.

**Conclusion:**

The burden of CVDRFs in stable outpatients is high across the national income categories in the AfME region, with HI countries showing a higher prevalence of CVDRFs. The high burden in lower income countries is associated with sub-optimal control of dyslipidemia and hypertension. Lowering the CVDRF burden would need specific public health actions in line with positive changes in the macro-economy of these countries.

**Trial registration:**

The ACE trial is registered under NCT01243138.

## Background

Conventional cardiovascular disease risk factors (CVDRFs) remain a main driver for the growing global burden of non-communicable diseases (NCDs) and premature cardiovascular mortality [1]. Worldwide, over 17 million deaths are now attributed annually to cardiovascular disease (CVD) [1]. Over the past few decades, the rate of cardiovascular mortality has declined markedly in many developed countries, likely due to reductions in CVDRFs and improved management of CVD [1]. On the other hand, the incidence of CVD has been increasing in many developing countries, where 80% of the global deaths from CVD are estimated to occur [2–4]. Notably, more than 60% of CVD, chronic kidney disease (CKD), and diabetes deaths in the developing world are attributable to preventable cardiometabolic risk factors, particularly hypertension, dyslipidemia, obesity, and diabetes mellitus, and nearly half occur prematurely in relatively young adults [2, 4].

While the term ‘developing countries’ often refers to countries of lower economic status, national incomes, and consequently national resources for CVD prevention, can vary considerably across these regions. In addition, drivers for CVD risk may vary by country income levels. For example, it is possible that rapid urbanization in Africa, coupled with limited resources to combat disease, may yield a different risk factor profile and CVD burden compared with countries experiencing rapid affluence and increased resources, such as the Arab Gulf states [5, 6]. A better understanding of the correlation between the macroeconomic spectrum in the developing world and CVDRF burden and control can help shape the efforts towards combating CVD burden in these countries [7]. In this report, we evaluate the prevalence of major CVDRFs – namely dyslipidemia, hypertension, obesity, diabetes mellitus and smoking – according to national income level.

## Methods

### Study design and population

The Africa Middle East Cardiovascular Epidemiological (ACE) study was a multicenter, multinational, cross-sectional, epidemiological study. The primary results of the ACE study were previously reported [8]. All participating sites had obtained relevant ethics/institutional review board or appropriate regulatory body approvals. The primary study was registered on clinicaltrials.gov (registration number NCT01243138). The ACE study and this income sub-analysis were conducted in 14 countries of the Africa and Middle East region across 94 general practice/outpatient rural and urban clinics between July 2011 and April 2012 [8]. Country selection was based on the availability of systemic epidemiological data, and site selection on the availability of expertise in clinical research and infrastructure to conduct clinical trials. Both urban and rural sites were included; rural sites were defined as being more than 50 km from an urban center and lacking easy access to commuter transportation [9]. To prevent selection bias, every fifth subject – male or female, over the age of 18 years, attending the clinic for any reason – was included in the ACE study [8]. Subjects with any life-threatening illness, or who were pregnant or lactating, were excluded.

Herein we evaluate the prevalence of major CVDRFs – namely dyslipidemia, hypertension, obesity, diabetes mellitus and smoking – according to national income level. The physician at the site performed the study evaluations, which included history taking, physical examination, and laboratory tests. The physicians at site performed all study evaluations during a single visit. However, non-fasting subjects at the visit were requested to return fasting at a second visit.

### CVD risk factor definitions

Dyslipidemia was defined as treatment with lipid-modifying medications or a fasting lipid profile measurement showing a high total cholesterol (≥240 mg/dL) or high low-density lipoprotein (LDL)-cholesterol level (≥100 to 160 mg/dL depending on risk factor classification), a low high-density lipoprotein (HDL)-cholesterol level (<40 mg/dL), a high triglyceride level (≥200 mg/dL), or a combination of these levels as per the National Cholesterol Education Program (NCEP) Adult Treatment Panel (ATP) III guidelines [10].

Hypertension was defined as evidence of treatment for blood pressure (BP) reduction or an arterial BP measurement ≥140/90 mmHg as defined by the European Society of Cardiology (ESC) CVD prevention guidelines [11]. BP was recorded as the higher of two consecutive measurements, taken once from each upper arm with the appropriate sized cuff and with the patient at rest and sitting for at least 5 min prior to measurement.

Body mass index (BMI) was defined as weight in kg divided by the square of height in meters. Obesity and abdominal obesity were defined as a BMI of ≥30 kg/m^2^ and a waist circumference of ≥94 cm in a male and ≥80 cm in a female, respectively, as per the International Diabetes Federation criteria [12].

Diabetes mellitus was defined as evidence of treatment with a hypoglycemic agent or fasting blood glucose measurement ≥126 mg/dL (7 mmol/L) as per the American Diabetes Association (ADA) guidelines [13].

Smoking was defined as current or past consumption of cigarettes, pipe, or water pipe (shisha). The use of medication – in particular antihypertensive therapy, lipid-modifying therapy, and therapy for diabetes (oral hypoglycemic agents and/or insulin) – was recorded.

### National income stratification

The national income categories were classified according to the World Bank atlas method, July 2011, which is based on the country’s Gross National Income (GNI) per capita in 2010. Low-income (LI) countries were those with GNI per capita of ≤ US $1005, lower-middle-income (LMI) countries with GNI per capita of $1006 to $3975, upper-middle-income (UMI) countries with a GNI per capita of $3976 to $12,275 and high-income (HI) countries with a GNI per capita of $12,276 or more [14]. Classification of countries according to national income is shown in Table 1.

**Table 1:** Classification of countries in this post hoc analysis of the cross-sectional Africa Middle East Cardiovascular Epidemiological (ACE) study conducted between July 2011 and April 2012, and stratified by national income group

Low-Income	Lower-Middle-Income	Upper-Middle-Income	High-Income
Kenya	Cameroon	Algeria	Kuwait
	Ghana	Jordan	Saudi Arabia
	Egypt	Lebanon	United Arab Emirates
	Nigeria	South Africa	
	Senegal	Tunisia	

### Statistical analysis

All efficacy summaries and analyses were conducted within the full analysis set population. Analyses were primarily descriptive in nature. No interim analyses were planned.

For dichotomous variables (prevalence and achievement of NCEP ATP III and ESC goals), the number and percentage of subjects were presented. Two-sided exact (Wilson Score test) 95% confidence intervals were presented for the percentage.

Continuous data (lipid parameters, BP parameters, and glucose) were summarized using descriptive statistics, including sample size, mean, standard deviation, median, minimum, and maximum. The differences across the income groups mentioned in this analysis were descriptive in nature and the study was not designed or powered to compare the prevalences/differences across the four income groups.

## Results

### Baseline characteristics by income group

This analysis of the ACE study evaluated 4378 outpatients. Overall, 31 subjects (0.7%) did not agree to continue to participate in the ACE study. Table 2 shows baseline characteristics of outpatients according to 4 national income categories: LI (*n* = 260, 6%), LMI (*n* = 1324, 30%), UMI (*n* = 1509, 35%) and HI countries (*n* = 1285, 29%). The cohorts across national income groups were relatively young, with mean age ranging between 42 and 49 years. The proportion of outpatients aged younger than 45 years ranged between 38%–57%, and the proportion aged 65 years or older ranged between 3%–15% (Table 2). The predominance of young outpatients was most notable in the LI cohort, where 57% were younger than 45 years and only 3% were aged 65 years and older.

**Table 2:** Patient characteristics in this post hoc analysis of the cross-sectional Africa Middle East Cardiovascular Epidemiological (ACE) study conducted between July 2011 and April 2012, stratified by national income group

Variable^a^	Low-Income *n* = 260	Lower-Middle-Income *n* = 1324	Upper-Middle- Income *n* = 1509	High-Income *n* = 1285
Age, n (%)				
18–44 years	149 (57.3)	624 (47.1)	579 (38.4)	661 (51.4)
45–64 years	104 (40.0)	581 (43.9)	703 (46.6)	542 (42.2)
≥65 years	7 (2.7)	110 (8.3)	227 (15.0)	81 (6.3)
Mean ± SD, years [Range], years	42.1 ± 12.3 [18–74]	45.2 ± 14.3 [18–110]	48.7 ± 15.2 [18–89]	44.6 ± 12.8 [18–88]
Male, n (%)	123 (47)	550 (42)	606 (41)	784 (62)
Female, n (%)	137 (53)	769 (58)	890 (59)	490 (38)
BMI (kg/m^2^), Median (25th, 75th percentile)	25.1 (21.8, 28.4)	26.8 (23.2, 31.4)	28.1 (24.8, 31.8)	29.1 (26.1, 32.7)
Waist circumference (cm), Median (25th, 75th percentile)	89.0 (81.0, 96.0)	91.3 (82.0, 102.0)	96.0 (86.0, 105.0)	95.0 (87.2, 105.0)
Total cholesterol (mg/dL), Median (25th, 75th percentile)	173.7 (146.9, 207.7)	184.0 (158.0, 217.0)	190.7 (162.0, 218.5)	182.0 (158.0, 208.0)
LDL-C (mg/dL), Median (25th, 75th percentile)	110.8 (81.1, 139.0)	116.0 (87.0, 146.3)	115.8 (92.7, 140.0)	107.0 (87.0, 131.3)
HDL-C (mg/dL), Median (25th, 75th percentile)	39.4 (31.3, 49.1)	47.0 (38.0, 57.0)	46.3 (38.0, 57.0)	45.0 (37.1, 53.0)
Triglycerides (mg/dL), Median (25th, 75th percentile)	97.4 (62.0, 127.7)	73.0 (45.0, 118.0)	86.0 (47.9, 136.0)	108.0 (62.0, 158.0)
Systolic BP (mmHg), Median (25th, 75th percentile)	133.0 (120.0, 146.0)	130.0 (120.0, 141.0)	130.0 (120.0, 140.0)	130.0 (120.0, 141.0)
Diastolic BP (mmHg), Median (25th, 75th percentile)	84.0 (74.5, 92.5)	80.0 (75.0, 90.0)	80.0 (76.0, 90.0)	80.0 (75.0, 90.0)
FPG (mmol/L), Median (25th, 75th percentile)	4.8 (4.1, 5.8)	5.0 (4.5, 6.0)	5.4 (4.8, 6.2)	5.6 (5.1, 6.6)

*BMI* body mass index, *BP* blood pressure, *FPG* fasting plasma glucose, *HDL-C* high-density lipoprotein cholesterol, *LDL-C* low-density lipoprotein cholesterol, *SD* standard deviation

^a^due to some information not being provided, not all combined percentages total to 100

### Prevalence of individual CVD risk factors by income group

**Fig. 1:**
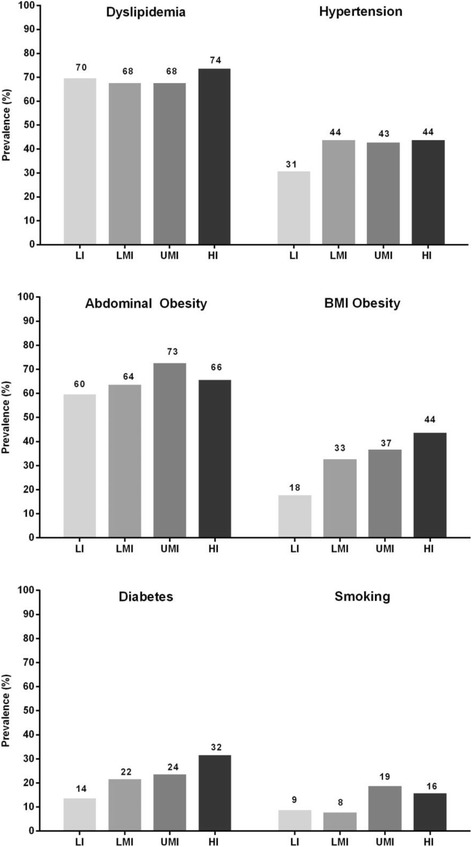
Prevalence of cardiovascular disease risk factors by national income group in this post hoc analysis of the cross-sectional Africa Middle East Cardiovascular Epidemiological (ACE) study conducted between July 2011 and April 2012. Countries in each income group are shown in Table 1. *HI* high income; *LI* low-income; *LMI* lower-middle-income; *UMI* upper-middle-income

**Fig. 2:**
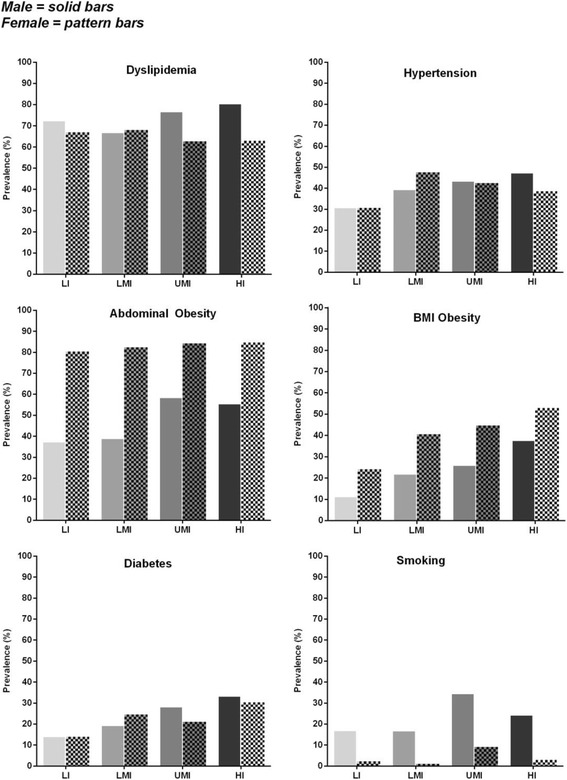
Prevalence of cardiovascular disease risk factors in this post hoc analysis of the cross-sectional Africa Middle East Cardiovascular Epidemiological (ACE) study conducted between July 2011 and April 2012, by gender and by national income group**.** Male outpatients (solid bars); female outpatients (patterned bars). Countries in each income group are shown in Table 1. *BMI* body mass index; *HI* high income; *LI* low-income; *LMI* lower-middle-income; *UMI* upper-middle-income

Dyslipidemia was the most prevalent risk factor, reported in nearly 70% of each population across the four national income categories (Fig. 1). While the highest prevalence of dyslipidemia was in the HI countries (74%), LI countries still had a significant burden, with 70% of patients having dyslipidemia (Fig. 1). By gender, the most notable difference in prevalence of dyslipidemia was in male vs. female patients from HI countries (81% vs. 63%; Fig. 2). When analyzing the individual components of dyslipidemia, HI countries had the highest median triglyceride level (108 mg/dL) but the lowest median LDL-cholesterol (107 mg/dL). However, all income groups had a median LDL-cholesterol above 100 mg/dL (Table 2). Low HDL-cholesterol was the most prevalent component of dyslipidemia across all income groups. Interestingly, the highest prevalence of low HDL-cholesterol was found in LI countries, where almost half of patients had low HDL-cholesterol levels (49%, compared with 28%, 28%, and 30% in LMI, UMI, and HI countries, respectively).

Median baseline SBP/DBP was similar across income groups, and ranged between 130.0–133.0/80.0–84.0 mmHg (Table 2). The prevalence of hypertension appeared to be lower in LI countries, and similar among other national income groups (Fig. 1). By gender, the prevalence of hypertension was generally similar across income groups (Fig. 2).

There was a progressive increase in BMI from a median of 25.1 kg/m^2^ in the LI group to 29.1 kg/m^2^ in HI group (Table 2). Consequently, BMI obesity was more than twice as common in HI compared with LI countries (44% vs. 18%; Fig. 1). When obesity was defined according to waist circumference (i.e. abdominal obesity), prevalence of obesity was much higher across all income groups; seen in approximately two-thirds of the overall cohort. Variability in the prevalence of abdominal obesity across national income levels was notably lower than that seen for obesity defined by BMI (Fig. 1). The higher prevalence of abdominal obesity compared with BMI obesity was most notable in LI countries, where abdominal obesity was three times more prevalent than a BMI ≥30 kg/m^2^ (60% vs. 18%). By gender, there was a consistently higher prevalence of obesity (defined by BMI or by waist circumference), in females compared with males across all income categories (Fig. 2).

Median fasting plasma glucose increased with higher national income, from 4.8 mmol/L in LI subjects to 5.6 mmol/L in the HI cohort (Table 2). This was paralleled by an increase in the prevalence of diabetes mellitus, which was seen in nearly one-third of the HI population; twice as common in HI countries compared with the LI population (32% vs. 14%) (Fig. 1). By gender, prevalence of diabetes was marginally higher in males vs. females in the UMI and HI countries; however, the prevalence of diabetes was marginally higher in females vs. males in the LMI countries (Fig. 2).

While smoking was the least prevalent of the CVDRFs considered, it affected over 10% of the overall study cohort and was notably more common in the UMI and HI countries than LI or LMI (Fig. 1). By gender, smoking prevalence was at least three-times higher in males compared with females across income groups, and this difference was most notable in the LMI and LI countries (Fig. 2).

### Clustering of CVD risk factors by income group

**Fig. 3:**
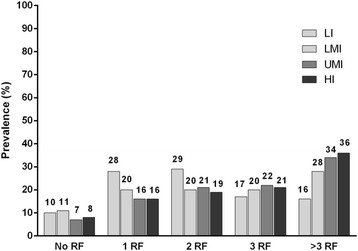
Number of cardiovascular disease risk factors stratified by national income group, in this post hoc analysis of the cross-sectional Africa Middle East Cardiovascular Epidemiological (ACE) study conducted between July 2011 and April 2012. Countries in each income group are shown in Table 1. *HI* high-income, *LI* low-income, *LMI* lower-middle income, *RF* risk factor, *UMI* upper-middle income

The majority of subjects within each of the income categories (approximately 9 out of 10) had at least one of the six modifiable CVDRFs analyzed in the ACE study (Fig. 3). Clustering of CVDRFs was more prevalent in HI countries, where more than one-third (36%) of subjects had at least four risk factors compared with 16% with four or more risk factors in LI countries (Fig. 3).

### Management of dyslipidemia and hypertension by income group

**Fig. 4:**
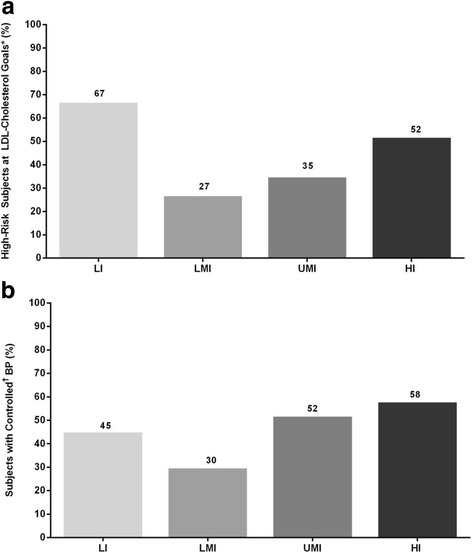
**a** Patients at high CV risk at LDL-cholesterol goals, and (**b**) blood pressure (BP) control in patients with a previous diagnosis of hypertension, in this post hoc analysis of the cross-sectional Africa Middle East Cardiovascular Epidemiological (ACE) study, conducted between July 2011 and April 2012, and stratified by national income group. *Patients with coronary heart disease (CHD) and CHD risk equivalents on lipid-lowering therapy were considered at goal if low-density lipoprotein (LDL) cholesterol is 100 mg/dL. ^†^Blood pressure (BP) was considered "controlled" if BP <140/90 mmHg. *HI* high-income, *LI* low-income, *LMI* lower-middle-income, *UMI* upper-middle-income

Within the overall cohort, 65%, 62%, 47%, and 34% of outpatients without a prior diagnosis of dyslipidemia were diagnosed with dyslipidemia when screened in the LI, LMI, UMI, and HI groups, respectively. Despite the high prevalence of dyslipidemia, only 4%, 5%, 16%, and 32% of outpatients were on lipid-regulating drugs in the LI, LMI, UMI, and HI groups, respectively. Between 27% and 67% of outpatients at high cardiovascular risk (i.e., with coronary heart disease [CHD] or CHD risk equivalents) and taking lipid-modifying medication met LDL-cholesterol targets as defined by NCEP ATP III guidelines, and this varied according to income level (Fig. 4a).

Overall, 8% to 13% of outpatients across income groups without a prior diagnosis of hypertension were diagnosed with elevated BP according to ESC criteria when screened. Among patients with a prior history of hypertension, an optimal BP reading was most common in HI countries (Fig. 4b).

## Discussion

Using a large, contemporary, cross-sectional database of stable patients attending outpatient clinics, we examined the prevalence and control of CVDRFs across a wide range of country-level income groups in the Africa and Middle East region. We found a significant burden of modifiable CVDRFs across national incomes in a relatively young population. While many of the risk factors were more prevalent in higher income countries, lower income populations still exhibited a significant burden of risk. Control of hypertension and treatment of dyslipidemia appeared sub-optimal across income levels, but were most notably poorer in lower income countries. Our findings complement the existing literature on cardiovascular epidemiology in the developing world, but provide novel insights into the cardiovascular disease risk burden and the impact of national income specifically in the Africa and Middle East region [15].

The observation of more prevalent CVDRFs with higher national income is consistent with recent global studies involving countries from the developing world. In the Prospective Urban Rural Epidemiologic (PURE) study, CVDRF burden increased steadily with higher national income [3]. Our study extends these findings in 12 other countries in the Africa and Middle East region that were not part of the PURE study, including countries in sub-Saharan and West Africa, where little systematic data exist on cardiovascular epidemiology. Notably, while CVDRFs had relatively lower prevalence in lower income countries relative to higher income countries, their absolute prevalence was still significant, particularly when coupled with fewer resources for managing the cardiovascular burden. Therefore, an equally important finding of the current study is the lower control of BP and utilization of lipid-lowering therapy seen in lower income countries. For example, although 70% of LI subjects had dyslipidemia, only 4% were taking lipid-lowering medication. Despite the relatively lower burden of risk, the sub-optimal resources to control such risk could translate into a higher disease burden. Although not reported in this study, poor control of risk factors may explain why the fatality rates of CVD are lowest in HI countries, intermediate in LMI and UMI countries, and highest in LI countries [3]. Lower fatality rates in HI countries are likely the result of greater use of preventive drugs, better control of hypertension, and lower smoking rates, in addition to wider access to a higher standard of care [3].

Deaths from CVD have been dramatically reduced in many ‘higher’ income countries owing to government policies that facilitate the adoption of healthier lifestyles and provision of equitable health care [16]. It is imperative that this favorable shift be sustained and replicated in lower- and middle-income countries. However, analysis of health systems shows that gaps in key elements of the health system, particularly at the primary care level, present obstacles to the provision of equitable health care for people suffering from NCDs. Health system strengthening – including adequate health financing, efficient governance, developed health workforce, robust health information, access to basic technologies and essential medicines, and health service delivery – should be a major focus of scaling up NCD prevention and control strategies [16]. Preventive strategies in this regard will be rewarded by an eventual decline in the rates of strokes and myocardial infarctions, which have become so prevalent in our communities.

Not only did we find high rates of individual CVDRFs, but of more concern is the tendency for these risk factors to co-exist (cluster) in many patients. Nearly three quarters of patients across income cohorts had at least two CVDRFs, approximately half had at least three, and approximately one-third of patients had at least four modifiable CVDRFs. Clustering of CVDRFs is well known [16] and combating such a multifaceted epidemic requires concerted multidisciplinary efforts involving key stakeholders, extending beyond patients and their physicians. National policies that focus on intelligent urban planning, evidence-based food policies, strict tobacco control, and innovative approaches to encouraging physical activity are therefore essential. Such policies should be an integral part of any social and economic development plan in order to manage (and preferably prevent) the associated increase in CVD burden. Urgent commitment from governments, policymakers, healthcare professionals, and all other stakeholders towards CVD prevention and promotion of healthy lifestyles is therefore essential, and economic development should be coupled with aggressive public health measures.

The present analysis is limited by the cross-sectional design of the study: patient selection is restricted to those attending outpatient clinics, reduced availability of expertise in these clinics; the reliance on one-time measurements of risk factors; and a lack of data on other variables such as social class and health insurance status that may affect cardiovascular risk and access to medications. Furthermore, given that this cohort had access to primary care, a selection bias may exist as these patients may be more affluent in comparison with the general population in the countries analyzed. The study therefore does not represent a population-based estimate of cardiovascular risk factor prevalence. Nonetheless, given the large sample size, potential selection bias is reduced. This study highlights the high prevalence of CVDRFs across all income groups in the Africa and Middle East region. Most of the published data for CVDRFs are based on studies in developed or developing (e.g., ‘high income’) countries. Although recent studies have provided new information, there is still a gap in the knowledge about risk factors and risk for CVD particularly in countries of lower- or middle-income [17, 18]. This study therefore adds key information to this knowledge base. In addition, it also highlights the importance of the opportunity for physicians in outpatient clinics to screen for CVDRFs using simple methods and to intervene appropriately.

## Conclusion

In conclusion, CVDRFs are widely prevalent across the national income spectrum in the Africa and Middle East region. While some CVDRFs and their clustering are more prevalent in higher income countries, lower income countries are not spared from the significant burden and have lower control rates for hypertension and dyslipidemia with appropriate medications. Economic development should be coupled with aggressive public health measures to limit the intake of salt, to encourage a healthier lifestyle and exercise in order to reduce the burden of obesity and diabetes mellitus, and to reduce the prevalence of smoking, thereby reducing the associated CVDRF burden. Some public health measures that could be considered require national strategies towards CVDRF management in the population, and ways for instituting protocols at primary care/outpatient clinics in order to identify CV risk early, and to manage CVDRFs optimally by allocating adequate resources. This applies equally to HI and LI countries as the CVDRF burden is high across national income groups; however different policies and resources will be required for different income countries in order to address the specific aspects of access to primary care.

## Abbreviations

ACE: Africa Middle East Cardiovascular Epidemiological
ATP: Adult Treatment Panel
BMI: Body mass index
BP: Blood pressure
CVD: Cardiovascular disease
CVDRF: Cardiovascular disease risk factor
ESC: European Society of Cardiology
GNI: Gross National Income
HDL: High-density lipoprotein
HI: High-income
LDL: Low-density lipoprotein
LI: Low-income
LMI: Lower-middle-income
NCD: Non-communicable disease
NCEP: National Cholesterol Education Program
PURE: Prospective Urban Rural Epidemiologic
UMI: Upper-middle- income

## References

[1] Roth GA, Forouzanfar MH, Moran AE, Barber R, Nguyen G, Feigin VL (2015). Demographic and epidemiologic drivers of global cardiovascular mortality. N Engl J Med.

[2] Global Burden of Metabolic Risk Factors for Chronic Diseases Collaboration (2014). Cardiovascular disease, chronic kidney disease, and diabetes mortality burden of cardiometabolic risk factors from 1980 to 2010: a comparative risk assessment. Lancet Diabetes Endocrinol.

[3] Yusuf S, Rangarajan S, Teo K, Islam S, Li W, Liu L (2014). Cardiovascular risk and events in 17 low-, middle-, and high-income countries. N Engl J Med.

[4] Yusuf S, Reddy S, Ounpuu S, Anand S (2001). Global burden of cardiovascular diseases: part I: general considerations, the epidemiologic transition, risk factors, and impact of urbanization. Circulation.

[5] Opie LH, Mayosi BM (2005). Cardiovascular disease in sub-Saharan Africa. Circulation.

[6] Rahim HF, Sibai A, Khader Y, Hwalla N, Fadhil I, Alsiyabi H (2014). Non-communicable diseases in the Arab world. Lancet.

[7] GBD 2013 Mortality and Causes of Death Collaborators (2015). Global, regional, and national age-sex specific all-cause and cause-specific mortality for 240 causes of death, 1990-2013: a systematic analysis for the global burden of disease study 2013. Lancet.

[8] Alsheikh-Ali AA, Omar MI, Raal FJ, Rashed W, Hamoui O, Kane A (2014). Cardiovascular risk factor burden in Africa and the Middle East: the Africa Middle East Cardiovascular Epidemiological (ACE) study. PLoS One.

[9] Teo K, Chow CK, Vaz M, Rangarajan S, Yusuf S (2009). The Prospective Urban Rural Epidemiology (PURE) study: examining the impact of societal influences on chronic noncommunicable diseases in low-, middle-, and high-income countries. Am Heart J.

[10] National Cholesterol Education Program (2002). Third report of the National Cholesterol Education Program (NCEP) expert panel on detection, evaluation, and treatment of high blood cholesterol in adults (Adult Treatment Panel III) final report. Circulation.

[11] Graham I, Atar D, Borch-Johnsen K, Boysen G, Burell G, Cifkova R (2007). European guidelines on cardiovascular disease prevention in clinical practice: executive summary: fourth joint task force of the European Society of Cardiology and Other Societies on cardiovascular disease prevention in clinical practice (constituted by representatives of nine societies and by invited experts). Eur Heart J.

[12] Alberti KG, Zimmet P, Shaw J, IDF Epidemiology Task Force Consensus Group (2005). The metabolic syndrome--a new worldwide definition. Lancet.

[13] American Diabetes Association (2010). Diagnosis and classification of diabetes mellitus. Diabetes Care.

[14] ChartsBin. Country Income Groups (World Bank Classification) [updated 1 July 2011]. Available from: http://chartsbin.com/view/2438 [Accessed 1 July 2011].

[15] Krishnamurthi RV, Feigin VL, Forouzanfar MH, Mensah GA, Connor M, Bennett DA (2013). Global and regional burden of first-ever ischaemic and haemorrhagic stroke during 1990-2010: findings from the global burden of disease study 2010. Lancet Glob Health.

[16] Yusuf S, Wood D, Ralston J, Reddy KS (2015). The World Heart Federation's vision for worldwide cardiovascular disease prevention. Lancet.

[17] Chen Z, Chen J, Collins R, Guo Y, Peto R, Wu F (2011). China Kadoorie Biobank of 0.5 million people: survey methods, baseline characteristics and long-term follow-up. Int J Epidemiol.

[18] Lawes CM, Rodgers A, Bennett DA, Parag V, Suh I, Ueshima H (2003). Blood pressure and cardiovascular disease in the Asia Pacific region. J Hypertens.

